# Performing populist leadership online: Discursive and multimodal construction of a shared social identity

**DOI:** 10.1111/bjso.70080

**Published:** 2026-04-06

**Authors:** Jenni Jaakkola, Inari Sakki, Eemeli Hakoköngäs, Jari Martikainen

**Affiliations:** ^1^ Department of Social Sciences University of Helsinki Helsinki Finland; ^2^ Department of Social Sciences and Business Studies University of Eastern Finland Kuopio Finland

**Keywords:** identity leadership, multimodal critical discursive psychology, populism, social identity, TikTok

## Abstract

Populist leaders are known for engaging supporters through compelling rhetoric, sparking debate about what persuasive strategies they use to mobilize voters. While research shows that leaders creatively frame their communication, the role of social media–especially its multimodal affordances–remains poorly understood. This study applies multimodal critical discursive psychology (MCDP) to examine the modalities used in TikTok videos of Finnish right‐wing populist politician Sebastian Tynkkynen. Using the integrative social identity model of populist leadership (ISIMPL), we identified eight discursive and multimodal strategies, through which Tynkkynen performs populistic identity leadership and constructs a shared identity: ‘performing populist prototypicality’ by emphasizing authenticity and ordinariness, ‘performing as the voice of the people’ through heroism and self‐sacrifice, ‘mobilizing a populist “us”’ through in‐group celebration and shared victimhood, and ‘othering the elite as “them”’ through blame and ridicule. These are accomplished through various discursive and multimodal resources, with co‐contextualization of elements playing a crucial role in creating an overall message. This study shows how multimodal communication enables populist politicians to innovatively perform leadership and construct shared identities online, enhancing understanding of the discursive and multimodal construction of populist identity leadership.

Throughout history, populist far‐right and right‐wing leaders like Hitler and Trump have gathered passionate and strongly committed supporters and successfully mobilized masses, sometimes resulting in dire consequences, such as the Capitol riot or the Holocaust (Haslam et al., 2020; Mols et al., 2023). However, the psychological and social processes that enable leaders to influence groups remain poorly understood. Recent research on populism increasingly focuses on the social identity perspective (Bos et al., 2020; Hameleers et al., 2019; Mols & Jetten, 2014, 2016; Ntontis et al., 2024; Obradović et al., 2020). The concept of social identity, based on the social identity approach (SIA), integrates social identity theory and self‐categorization theory (Tajfel & Turner, 1979; Turner et al., 1987), suggesting that individuals base their self‐concept on valued group memberships. This involves a tendency to construct the social world by fostering positive evaluations of the in‐group and negative evaluations of the relevant out‐group, thereby influencing collective behaviour. Since populism operates through an antagonistic logic, distinguishing the good ‘us’ from the bad ‘them’ (Laclau, 2005; Mudde, 2004), it can be seen as a concrete manifestation of in‐group and out‐group thinking. Thus, SIA offers a novel perspective by focusing on the social and psychological group processes that populist radical right parties (PRRPs) seek to exploit when mobilizing supporters (Mols et al., 2023).

Populist leaders can thus leverage social identities for political purposes. Alongside leaders' actions (performances, style), the language used is crucial (Mols et al., 2023), indicating that the construction of populist social identity is inherently discursive. Jurstakova et al. (2022) further elaborated that leaders craft social identities not only through rhetorical (language) but also performative (rallies, demonstrations) means to define ‘us’ and foster a sense of empowerment among supporters. For example, Trump utilized confrontation in his speech before the Capitol attack, distinguishing morally good ‘American patriots’ from bad Democrats to foster a sense of community among supporters and an obligation to act (Ntontis et al., 2024). In the more distant past, Hitler succeeded in forming a disciplined group and united identity through theatrically staged rallies (Haslam et al., 2020).

However, political discourse is increasingly shifting online, providing new spaces and tools for constructing uniting identities (Gal et al., 2016; Hakoköngäs et al., 2020), changing its logic and introducing new forms of political expression emphasizing visual (Luhtakallio & Meriluoto, 2022) and multimodal (Pettersson et al., 2023) content. The use of online arenas for conveying ideological messages and mobilizing supporters has therefore enhanced multimodal persuasion, meaning that communication combines, for instance, verbal, visual and sonic elements (Kress, 2011). Together, these elements can create emotionally powerful, persuasive and engaging political arguments.

PRRPs have successfully utilized social media and its multimodal affordances to construct a shared populist identity through antagonistic divisions between ‘us’ and ‘them’ (Pettersson et al., 2023) and to mask hateful messages in visuals or humour (Sakki & Martikainen, 2021). However, multimodal research has not yet been connected to populist identity leadership, despite the growing prevalence of such communication on social media. This research gap is significant because social media enables political leaders to communicate with their audiences more directly and interactively, bypassing the journalistic gatekeeping of traditional media (Moffitt, 2016). As a result, it gives them greater control over the content they present through personalized and innovative multimodal posts. Through these affordances, leaders can express populist ideology more freely and creatively construct a shared identity with their followers. This study aims to fill the gap in the literature and contribute to the social psychology of populist identity leadership, particularly in the context of social media, by demonstrating how politicians use multimodal discursive strategies to perform leadership and reinforce a shared identity online. These insights may reveal new mechanisms of influence and clarify how emerging digital platforms function as arenas of political persuasion.

## POPULIST SOCIAL IDENTITY LEADERSHIP

As stated, leaders play a central role in mobilizing groups (Haslam et al., 2020). Haslam et al. (2020) conceptualized this process through the identity leadership model (ILM), rooted in SIA, emphasizing leadership as an identity‐based process where influence is granted through alignment with a shared identity. Theory identifies four components that enhance a leader's effectiveness to shape actions and values of the group members' shared self‐understanding. Effective leaders must (1) be seen as *in‐group prototypes*, one of ‘us’, embodying characteristics that distinguish the in‐group from the out‐group; (2) work for the group as an *in‐group champion* through identity advancement and supporting its goals; (3) actively foster a shared sense of ‘us’ by defining the group's social identity as *entrepreneurs of identity* and determining members of the group, content (norms, values) and prototypicality; and (4) act as *embedders of identity* by mobilizing ‘us’ towards shared goals, turning the vision of social identity into reality and bringing the group together through participatory ritualized performances of identity (Haslam et al., 2020; Mols et al., 2023).

However, leadership is not a one‐sided process. For this reason, Haslam et al. (2022) further developed a dual‐agency model of leadership, where collective action emerges from collaboration between leaders and followers who identify with the same social group. Here, leaders foster a sense of ‘us’ and articulate and define in‐group goals, while followers affirm the shared identity and demonstrate loyalty by acting creatively to realize these aims.

Furthermore, Uysal et al. (2022) considered these models as suitable for populists leaders and extended them to describe how populists function as identity leaders. They proposed an intergrative social identity model of populist leadership (ISIMPL) framework to complement the image. This model suggests that populism echoes the same antagonistic logic defined in SIA through a *Manichean worldview* that divides society into ‘us’ and ‘them’ (Mudde & Kaltwasser, 2017). The success of populist leaders lies in their ability to construct a positive in‐group identity for ‘the people’ while portraying out‐groups as enemies or threats (Bos et al., 2020; Mols & Jetten, 2016; Mudde, 2004). This binary mirrors a belief in the *people's will* and *anti‐establishment sentiments*, suggesting that elites are disconnected from the needs of ordinary people, failing to represent them (Uysal et al., 2022).

In this context, populist leadership is an active process, requiring leaders to perform in a certain way to underscore the significance of the people and foster an in‐group identity. Goffman's dramaturgical theory and the concept of self‐presentation elucidate this process, indicating that individuals tend to emphasize an idealized image to audiences (Goffman, 1959). On social media, this is referred to as strategist profile work – presenting a curated and consistent image by choosing what to share (Enli, 2015; Uski, 2015). Populism is indeed viewed as fundamentally performative, with the leader as a key actor and social media serving as an ideal platform for this performance (Moffitt, 2016). According to ILM, effective leaders should emphasize commonalities with their group (Haslam et al., 2020; Rooyackers & Verkuyten, 2012). In populism, leaders must convince voters that they are ordinary citizens like themselves (Reicher & Haslam, 2017).

In addition to appearing as one of the people, based on ILM theory, populist leaders must present themselves as the people's *representative*, acting on behalf of the virtuous in‐group (Haslam et al., 2020; Rooyackers & Verkuyten, 2012), as belief in the ‘people's will’ is central (Mudde & Kaltwasser, 2017). By aligning their concerns with the people, populist leaders reinforce a sense of common membership and claim to represent the people, unlike mainstream politicians labelled as internal enemies (Mols & Jetten, 2014; Sakki & Pettersson, 2016). At its best, social media can, amplify this, as interactive platforms and real‐time updates enable direct engagement with the people and on their behalf (Moffitt, 2016).

The mobilizing power of populist leaders lies also, as outlined by ILM, in their ability to foster a *sense of ‘we‐ness’* among the in‐group, building a shared identity by actively defining what ‘us’ means (Reicher & Hopkins, 2001). This means presenting ‘the people’ in a positive light and emphasizing their moral superiority (Ntontis et al., 2024), which can today be done through innovative, multimodal strategies on social media (Pettersson et al., 2023; Sakki & Martikainen, 2021). This also involves acting as *identity impresarios*, creating participatory, performative rituals that reflect the in‐group's vision (Haslam et al., 2020; Jurstakova et al., 2022), which can now also take place virtually.

Populist leaders also use narratives of shared victimhood and a threatened nation as a strategic tool of identity leadership, portraying the in‐group as morally good and innocent victims wronged by a threatening out‐group (Mols & Jetten, 2014, 2016; Ntontis et al., 2024; Reicher & Uluşahin, 2020). This identity threat unites the group, as it draws on the nostalgic core idea of populism that the once‐powerful group lost its position, thereby fuelling resentment and legitimizing actions to reclaim this lost status (Mols & Jetten, 2014).

Constructing morally good but victimized shared identity also involves identifying a culprit for the group's suffering, as it helps unite supporters under a shared identity (Hochschild, 2018; Mols & Jetten, 2016). This happens often through blaming discourse (Hameleers et al., 2017), moralizing out‐groups as corrupt or dangerous (Ntontis et al., 2024). Blame is typically placed on elites, societal out‐groups like immigrants, or other perceived ‘enemies within’ (Hameleers et al., 2017; Hirsch et al., 2021; Sakki & Martikainen, 2021; Sakki & Pettersson, 2016). Social media relies on emotionality and dramatization (Moffitt, 2016) but through multimodal elements, it is also possible to “mask” this disparaging discourse in a socially acceptable form (Pettersson et al., 2023; Sakki & Martikainen, 2021).

While an increasing number of studies have approached populism through the lens of social identity (e.g., Bos et al., 2020; Hameleers et al., 2019; Mols & Jetten, 2014, 2016), less attention has been given to how populist politicians perform identity leadership and construct shared identities on social media. Building on research that underscores the central role of leaders in mobilizing social identity, this article addresses this gap by exploring how populist leaders enact identity leadership through discursive multimodal strategies in online environments. Drawing on the ISIMPL framework (Uysal et al., 2022), it examines how identity leadership is performed and constructed discursively and multimodally.

Empirically, this study provides new insights into the role of multimodal populist identity leadership and social identity in political persuasion within online communication. Theoretically, it applies social psychological theories of populist identity leadership in the new context of social media. Methodologically, it combines rhetorical and discursive qualitative methods with multimodal approaches, employing multimodal critical discursive psychology (MCDP).

## MCDP ANALYSIS OF TIKTOK VIDEOS

This article examines the TikTok videos of Sebastian Tynkkynen, a politician from the Finnish PRRP Finns Party (FP; *Perussuomalaiset*), who has been active on social media and gained national popularity with 135,600 followers on TikTok by the analysis date (November 2024). He was a member of Parliament from 2019 to 2024, currently serves as the third vice chairperson of the FP and was elected to the European Parliament in 2024. When Tynkkynen joined the FP, it had already risen from a small agrarian party to one of the largest in the Finnish Parliament (Herkman, 2024), transforming into a nativist PRRP (Jungar & Jupskås, 2014). He has used provocative rhetoric on key FP issues (Herkman, 2024; Pettersson, 2020), including anti‐immigration, anti‐environmental policies (Lonnqvist et al., 2020) and EU‐scepticism (Herkman, 2017, 2024). He has also openly discussed his Pentecostal upbringing; homosexuality; and prosecution for hate speech against Muslims, which he, however, denies committing (Pettersson, 2020).

After securing the TikTok research API, a total of 277 of Tynkkynen's publicly published TikTok videos between 28 December 2019 and 2 April 2023 were collected. The research material covers all his videos published on the platform before the 2023 Finnish parliamentary elections, reflecting a time when the FP was in opposition preparing for the elections. Research shows that TikTok influenced voter behaviour, with its usage correlating with support for the FP in elections (Tukiainen et al., 2023), making it crucial to analyse the online strategies employed to gather a follower base. Using Tynkkynen as an example, we aim to understand how populist leadership and social identity are performed on social media through multimodal means. We utilize the MCDP approach (Pettersson & Martikainen, 2025), integrating critical discursive psychology (CDP; Edley, 2001; Wetherell, 1998) and multimodal discourse analysis (MDA; Kress, 2011; O'Halloran, 2008) to analyse how various discursive and multimodal components (verbal, visual, sonic, textual, digital) co‐produce meanings in political discourse (Kilby & Lennon, 2021; Pettersson et al., 2023).

CDP, which has its roots in social constructionism, is a methodological framework that centres around language and discourse (Edley, 2001). It asserts that people use language to construct versions of the social world while also stressing that the language used is itself constructed by people (Potter, 1998), thus focusing on language as a form of action itself (Potter & Wetherell, 1987). CDP integrates discourse and rhetorical psychology (Billig, 1987; Potter & Wetherell, 1987), enabling detailed examination of discourses and rhetorical strategies used to construct meanings and how these constructions are linked to broader societal discussions and tensions (Edley, 2001). It approaches discourse at the intersection of the societal ‘macro’ and individual ‘micro’ levels considering both the immediate social context and the broader socio‐political context (Edley, 2001), making it as a useful tool to examine political discourse on social media. In this study, we analyse Tynkkynen's TikTok communication from a critical discursive psychological perspective (Edley, 2001; Wetherell, 1998), using a three‐step process that examines the content, form and function of discourse (Sakki & Pettersson, 2016). This pragmatic CDP approach complements Wetherell's (1998) widely used synthetic formulation by providing a structured, stepwise procedure while situating micro‐level rhetorical practices within broader contemporary political and digital contexts. It allows us to examine how Tynkkynen constructs and mobilizes (social) identities (i.e., subject positions) and their ideological and political functions. The approach has proven useful for analysing political discourse in both online and offline contexts (Pettersson et al., 2023) and multimodal political discourse (Pettersson et al., 2023; Pettersson & Martikainen, 2025).

By examining TikTok videos as a whole, we expand the analysis from language to broader multimodal means of political discourse. Rooted in social semiotics, MDA (Kress & van Leeuwen, 2021) examines how multiple simultaneous semiotic resources (e.g., verbal, visual) are combined to create meanings and enhance the persuasive potential of messages, thereby enabling an exploration of how political discourse draws from socially shared meanings grounded in the socio‐cultural context (Kress, 2011; O'Halloran, 2008). To integrate CDP and MDA, we employ the systemic functional approach (Halliday, 1978, 1994), focusing on the composition of multimodal resources (content), the meanings they produce and their interaction with viewers (form and function) in online political discourse and exploring how similar elements produce consistent meanings to reinforce meaning potentials (co‐contextualization) or how divergent elements generate differing meanings to create tensions (re‐contextualization; O'Halloran, 2008; Royce, 2007). This expands the CDP's three‐phase analytical procedure (Pettersson & Martikainen, 2025), incorporating and providing tools to study the multimodal grammar of communication in a particular social, political and cultural context. Despite the different theoretical foundations of MDA and CDP, the combination is successful because both approaches view discourse as a fundamentally social and functional process (Kilby & Lennon, 2021; Pettersson & Martikainen, 2025).

In the first stage of analysis (Pettersson & Martikainen, 2025), we watched all 277 videos (0.10–10 min long, often under 3 min) multiple times to deeply understand the overall *content*. This enabled us to divide videos into smaller analytical units based on their identified main *content* considering all the components. Next, we conducted rough multimodal transcriptions for each video, and detailed multimodal transcriptions for selected videos from each analytical unit, considering spoken, visual, textual, musical and digital elements and their interplay (Pettersson & Martikainen, 2025) and focusing on both the *content* and *form* of the multimodal narrative. Following CDP, we examined both *content* (what is said) and *form* (how it is said), focusing on rhetorical strategies and discursive resources used (Potter, 1996; Potter & Wetherell, 1987) to construct populist leadership. This reveals that by employing certain *content* and *forms* of expression, specific meanings are constructed, which in turn creates multimodal rhetorical strategies. Finally, we explored the videos' ideological and political *functions* in a broader argumentative socio‐political context. We focused on the interplay of modalities and the social functions they serve (O'Halloran & Lim, 2014; Sakki & Pettersson, 2016) and aimed to understand what *functions* are intended to be realized through the identified strategies, recognizing modalities as semiotic resources with specific meaning potentials that become emphasized when combined (O'Halloran, 2008). Specifically, we analysed how the videos construct shared populist identity and persuade audiences in light of ISIMPL (Uysal et al., 2022).

## MULTIMODAL ANALYSIS OF MOBILIZING POPULIST IDENTITY

Based on the MCDP analysis, we identified eight multimodal and discursive strategies Tynkkynen uses to perform identity leadership and construct shared identity. These strategies form four functions: (1) performing populist prototypicality; (2) performing as the voice of the people; (3) mobilizing a populist ‘us’; and (4) othering the elite as ‘them’ (Table 1).

**TABLE 1:** Content, form and function of Tynkkynen's TikTok videos in relation to populist leadership and common identity.

Modalities	Multimodal discursive strategies	Populist identity leadership (function)
(content + form)		
*Spoken*: jokes, talks lightly about everyday life *Textual*: summarizes key points, humorous narrative *Visual*: amusing moments, everyday action and objects, various locations, shared entertaining videos, checkered shirt, smiles, colours *Music*: cheerful music, well‐known songs *Digital*: emojis (laughing), funny gif	Ordinariness (*by entertaining*; *n* = 27)	Performing populist prototypicality (*n* = 36)
*Spoken*: speaks emotionally about feelings, difficulties and past; dialect *Textual*: creates emotional narrative *Visual*: childhood pictures, closeups, wistful facial expressions, tears *Music*: powerful emotional music, meaningful lyrics *Digital*: layered videos, alternating images	Authenticity (*by expressing emotions*; *n* = 9)	
*Spoken*: discusses requested political topics, seeks followers' opinions *Textual*: key points *Visual*: serious expression, straight back, in parliament, films other members of parliament *Music*: calm, chivalric *Digital*: red and black borders	Heroism (*by professionalism*; *n* = 82)	Performing as the voice of the people (*n* = 87)
*Spoken*: states self‐sacrifices *Text*: subtitles, creates narrative *Visual*: speaks to the camera, appealing eye contact, moments of sacrifice, serious expression *Music*: calm, absent	Self‐sacrifice (*by extremes*; *n* = 5)	
*Spoken*: delivers information about group values, praises followers, mobilizes to act *Textual*: summarizes key points, narrative praising the group *Visual*: speaks to the camera, smiles, persuasive gestures, colourful *Music*: energetic, familiar songs, meaningful lyrics *Digital*: emojis (heart, Finnish flag), videos and pictures sent by followers	Celebrating ‘us’ (*by informing, mobilizing and rejoicing*; *n* = 90)	Mobilizing a populist “us” (*n* = 103)
*Spoken*: describes mistreatment towards ‘us’ *Textual*: key points *Visual*: speaks to the camera, situations where mistreatment occurs, dark, serious expressions, furrowed brows *Music*: absent *Digital*: added images (headlines revealing bullying)	Shared victimhood (*by dramatizing*; *n* = 13)	
*Spoken*: blames ‘them’, moral labelling *Textual*: key points *Visual*: speaks to the camera, serious expression *Music*: absent *Digital*: emojis (angry), coloured borders, pictures and videos of other politicians	Blaming (*by moral labelling*; *n* = 19)	Othering the elite as ‘them’ (*n* = 51)
*Spoken*: ridicules ‘them’ ironically *Textual*: key message, joke narrative *Visual*: speaks or performs to the camera, clips of political opponents speaking, meme‐like, colours, expressions *Music*: cheerful *Digital*: emojis (cry‐laugh), filters	Ridiculing (*by irony*; *n* = 32)	

## PERFORMING POPULIST PROTOTYPICALITY

The first identified *function* of Tynkkynen's videos is ‘performing populist prototypicality’ (in 36 videos, hereafter, the number of videos is in parenthesis), where the leader presents himself as the prototype of the in‐group, emphasizing shared characteristics that distinguish from out‐groups (Haslam et al., 2020). In this case, populist prototypicality is constructed through two multimodal and discursive *strategies*: (1) performing ordinariness and (2) authenticity.

### Ordinariness

Populist leaders must convince voters they are not part of the political elite (Reicher & Haslam, 2017). Instead, they seek to present themselves as ordinary people (Moffitt, 2016; Rapley, 1998), appearing relatable and ‘human’ (Wood et al., 2016). This includes down‐to‐earth qualities and doing everyday activities (Enli, 2025), facilitating identification.

Through semantically congruent modalities that *co‐contextualize* each other (O'Halloran, 2008; Royce, 2007), the videos with entertaining content (27; Beck, 2024) construct Tynkkynen as an ordinary entertainer rather than a formal politician (Rapley, 1998), thereby positioning his prototypicality as an ordinary citizen (Haslam et al., 2020). He jokes or lightly discusses daily life to the camera (12), while text summarizes jokes or mundane topics (20). Many videos lack narration, relying on other multimodal elements. Visually, he films amusing moments featuring everyday actions/objects and TikTok challenges in various locations (10) or shares entertaining meme‐like videos (5) similar to other TikTokers, communicating relatability (Enli, 2025). He often wears a red checkered shirt (17); choosing casual attire over a formal suit highlights the divide between ordinary people and the elite (Grabe & Bucy, 2009; Szebeni & Salojärvi, 2022). The positive atmosphere is reinforced by bright, saturated colours, colourfulness (Kress & van Leeuwen, 2021) and his smiling demeanour (13; Feng & O'Halloran, 2012). Also, added elements construct a positive tone. Music is frequently used; cheerful songs (16), including well‐known pop or theme songs, resonate with familiarity and further uplift the mood (Abidin, 2021). Tynkkynen also plays with digital elements abundant in videos. Emojis (8; e.g., laughing) and gifs add playful visual appeal, reminiscent of content typical of social media users.

### Authenticity

Populist politicians also seek authenticity (Enli, 2025; Szebeni & Salojärvi, 2022; Wodak, 2020) to appear as genuine citizens, contrasting them with the untrustworthy and distant elite (Enli, 2025). Performative strategies like consistency, spontaneity, confessions and imperfection enhance perceived authenticity, conveying trustworthiness and sincerity (Enli, 2025). Importantly, public and private performances must align (Goffman, 1959; Shane, 2018).

Videos position Tynkkynen's authenticity through emotional and personal content (9; Enli, 2015, 2025). Tynkkynen openly discusses his feelings, humble background and past challenges (5) and uses dialect (Enli, 2015; Pettersson et al., 2023) or emotional narrative in the text (4) to communicate emotional appeal and reveal personal details. Visually, the videos include childhood pictures, closeups of Tynkkynen and wistful expressions with tears (2), adding emotional resonance and revealing his genuine feelings. Emotionally powerful music and lyrics reinforcing the narrative (4) further deepen the impact (Abidin, 2021). Additionally, digital techniques, like layered videos or alternating images (3), juxtapose Tynkkynen's modest past with the present, increasing the emotional value of his achievements (Enli, 2025). Again, semantically congruent modalities are set in *co‐contextualizing* relations to reinforce each other's meaning potentials (O'Halloran, 2008; Royce, 2007), enhancing emotional depth and openness, thereby reinforcing an authentic image of Tynkkynen revealing his true self (Enli, 2025) as a genuine prototypical citizen (Haslam et al., 2020). The multimodal construction of authenticity is demonstrated in the following example (Table 2).

**TABLE 2:** Multimodal Transcription of Video 271.

Spoken	Visual	Text	Digital
*0:00–0:10* ‘Well, listen up. Poor can take power from the rich. Here we come. Fuck the whole state power.’ (laughs) ‘Hello’	Tynkkynen films himself walking into Parliament wearing a suit. He opens the plenary hall door, walks in, smiles and greets a passerby	‘1. and last time in the chamber’	Two layered videos. The upper video plays
*0:11–0:15* ‘Let's see… Now I just need to find my own seat’	Tynkkynen looks around		
*0:34–0:43* ‘Let's get to work for the good of Finland and fight against all kinds of injustice and unfairness’	Tynkkynen speaks and fixes his hair	‘1. Time in the chamber’ ‘Last time’	
*0:44–0:52* ‘It feels really strange to be here now. Like, I've watched this activity here so much, to be honest, it's like a circus here’	Tynkkynen looks around the chamber		
*0:59–1:27* ‘Well. It's time to walk out that door and leave the plenary hall for the last time. Four years have passed since I walked in here, completely inexperienced in life, into this grand hall, wondering how it's possible to come represent everyone in Finland from such circumstances.’ (Talking in the background.)	Tynkkynen films himself in the plenary hall, wearing a suit. With tears in his eyes, he adjusts his glasses, looks at the camera, occasionally glancing around		The lower video plays
*1:28–1:50* ‘But it happened, and thanks to everyone who gave me the opportunity to be your voice. I can still say it feels really strange to have been here’	Tynkkynen sighs and looks into the camera. He furrows his brows. Lower jaw trembles. He wipes a tear		

*Source*: https://www.tiktok.com/@sebastiantynkkynen/video/7215996858181733658 (29 March 2023, 4:47 min).

The video consists of two digitally layered clips of Tynkkynen filming himself, with a self‐recorded format emphasizing authentic emotions and an amateurish feel (Ross & Rivers, 2018). The text summarizes the events; ‘1. and last time in the chamber’ reinforcing the nostalgic tone (Mols & Jetten, 2014). In the first clip, Tynkkynen arrives in the plenary hall defiantly stating, ‘Well, listen up, poor can take power from the rich. Here we come. Fuck the whole state power’ (0:00–0:10). Impoliteness (Wodak et al., 2021) and swearing conveys identification with authentic people (Montiel et al., 2022) as well as positioning himself as a ‘poor’ citizen against the powerful wealthy (Mols & Jetten, 2016).

**FIGURE 1:**
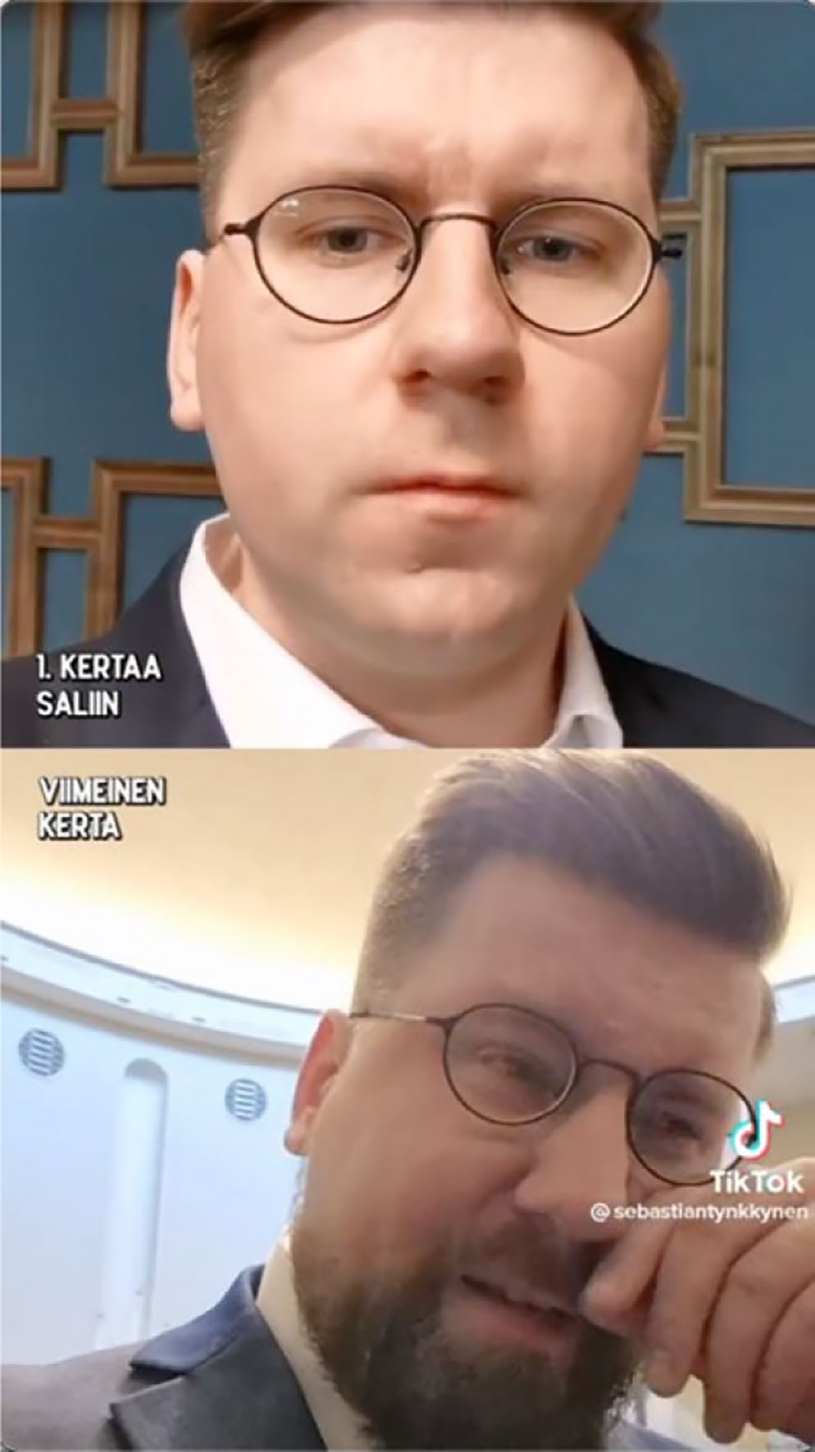
Screenshot of Tynkkynen's TikTok Video 271.

In the second clip, Tynkkynen reflects on his journey: ‘Four years have passed since I walked in here, completely inexperienced in life, into this grand hall, wondering how it's possible to come represent everyone in Finland from such circumstances’ (0:59–1:27). He continues expressing gratitude with a broken voice: ‘thanks to everyone who gave me the opportunity to be your voice’ (1:28–1:50). Emphasizing similarity to people, he discusses his humble past and uses demotic speech (Rapley, 1998; Rooyackers & Verkuyten, 2012). He gazes closely at the camera, wiping tears with a vulnerable demeanour (Feng & O'Halloran, 2012; Figure 1), indicating authentic emotions (Enli, 2015; Wood et al., 2016).

Semantically congruent visual (closeup, tears), spoken (‘fuck’, broken voice, gratitude, dialect, humble past) and digital (layered clips, self‐recorded) modalities are set into *co‐contextualizing* relations (O'Halloran, 2008; Royce, 2007), together enhancing emotional impact through aggression and modesty, thereby reinforcing an authentic image as ‘one of us’.

## PERFORMING AS THE VOICE OF ‘THE PEOPLE’

The second identified *function* is ‘performing as the voice of “the people”’ (86), where the leader demonstrates commitment to promote the interests of the in‐group (Haslam et al., 2020) – in this case, embodying the common people. This is constructed through two multimodal and discursive *strategies*: (1) heroism and (2) self‐sacrifice.

### Heroism

While performing ordinariness, populist leaders also need to appear as heroes – suited and ready to lead, confront enemies and restore a glorious future (Mols & Jetten, 2014; Reicher & Hopkins, 2001). This means performing as exceptional (Moffitt, 2016; Schneiker, 2020) or ‘strongmen’ (Mudde & Kaltwasser, 2017).

The videos position Tynkkynen as a heroic people's spokesperson, underlining professionalism (82). Tynkkynen substantively discusses requested political topics (70; e.g., gas prices), highlighting his parliamentary work. He also seeks followers' opinions (7), communicating responsiveness to the in‐group's will (Mudde & Kaltwasser, 2017). Added textual elements summarize key messages, reinforcing the importance of the spoken topics (43). Visually, a solemn manner and upright posture in parliament (51) underscore his role as a professional representative (Mudde & Kaltwasser, 2017). Scenes of other MPs speaking inconsistently and focusing on chatting or their phones during his speech (14) contrast their selfish behaviour with his professionalism. Music plays a key role in reinforcing this perception: calm music (24) enhances professionalism, while powerful, chivalric music (7) emphasizes heroism (Machin, 2010). Also, digitally added borders in red and black (24), associated with power and seriousness (Kress & van Leeuwen, 2021), highlight authority. Thus, congruent modalities are set into *co‐contextualizing* relations (O'Halloran, 2008; Royce, 2007), complementing meanings enhancing professionalism, thereby communicating a heroic image of (Schneiker, 2020) as the voice of the people (Haslam et al., 2020). Instead, the contrasting visual modalities of others serve as *re‐contextualizing* elements, depicting ‘them’ as unprofessional.

### Self‐sacrifice

For leaders, self‐sacrifice and a willingness to do anything for the group are crucial as it demonstrates group‐orientedness and true dedication, fosters loyalty and trust, and strengthens their position (van Knippenberg & van Knippenberg, 2005). In populism, defending the people's sovereignty at any cost is important (Mudde & Kaltwasser, 2017).

Semantically congruent modalities *co‐contextualize* each other and reinforce meanings (O'Halloran, 2008; Royce, 2007), all underlining the extreme actions taken with serious undertones (5), thereby constructing Tynkkynen as a self‐sacrificing representative (Rooyackers & Verkuyten, 2012), positioning him as a voice of the people (Haslam et al., 2020). Tynkyknen verbally claims to have helped people to the extent of receiving fines and pushing his body to its limits (4). The textual narrative emphasizes self‐sacrifice (4). Visually, the imagery consists of three key scenes: Tynkkynen justifying his actions by speaking in front of the camera with appealing eye contact (3), facing charges in court (1), and delivering an over 8‐h speech in parliament (2) with a grave expression and bowed head, proving suffering and sacrifices. While calm pop music plays in the videos of him receiving charges and speaking in parliament at the limits of his body (2), music is absent when addressing the audience, further increasing the seriousness (Machin, 2010). The following example from a campaign video illustrates how self‐sacrifice is constructed through multimodality (Table 3).

**TABLE 3:** Multimodal transcription of Video 274.

Spoken	Visual	Music	Text
*0:24–0:41* ‘I decided not to wait for these four years, because even without power, one can influence by voicing the concerns of the people and ensuring that they do not forget the decisions made when power is distributed in the next elections. I decide to use the voice given to me all the time and everywhere’	Short clips: Tynkkynen carrying filming equipment and speaking at events, protests, interviews, and to the camera. The video transitions to a cityscape aerial view and moves to Tynkkynen speaking to the camera and at the speaker's podium	Calm instrumental music	
*0:42–0:51* ‘I took on fines of over ten thousand euros for bringing the distress of citizens to light and spoke against the EU recovery package until my body gave in’	Tynkkynen films himself, with people in the background. Next, he sits in court surrounded by photographers and speaks at a campaign event and in the plenary hall leaning on the podium		
*0:52–1:09* ‘I have learned that it doesn't take money to open your mouth. That's why my budget for these elections is 0€. If you want a voice that will be heard even after the polling booths, advertisements, and politicians are gone, I'm at your service’	The pace of the video clips accelerates, and ageing Tynkkynen is filmed speaking to the camera in different backgrounds wearing different clothes. The text ‘Reserve your advertising space on this display’ appears on an advertising board, then fades to black		‘SEBASTIAN TYNNKKYNEN. Your voice after elections’

*Source*: https://www.tiktok.com/@sebastiantynkkynen/video/7217195004920384795 (2 April 2023, 1:09 min).

**FIGURE 2:**
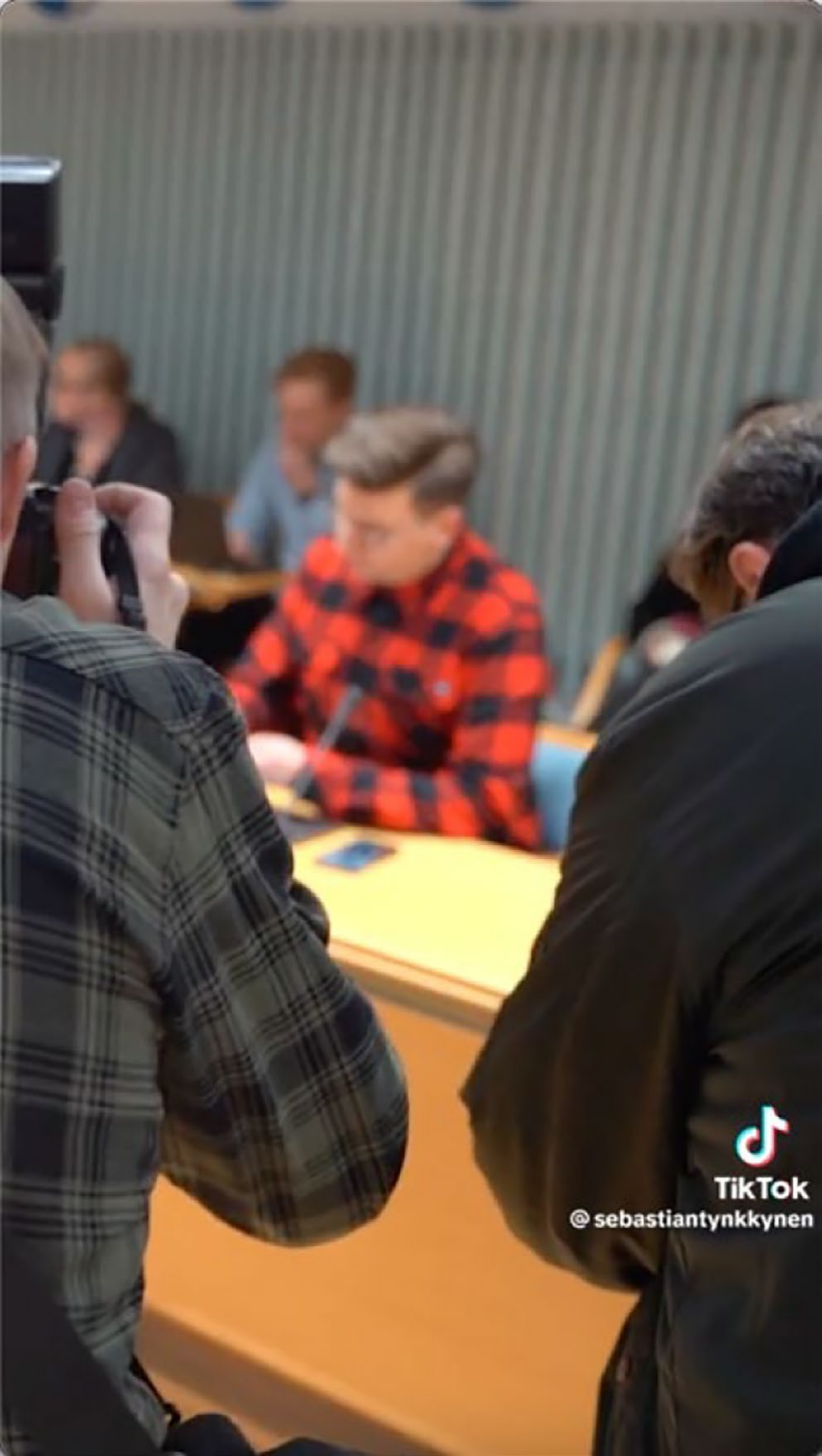
Screenshot of Tynkkynen's TikTok Video 274.

At the beginning, Tynkkynen states in a rebellious tone: ‘Even without power, one can influence by voicing the concerns of the people… I decided to use the voice given to me all the time and everywhere’ (0:24–0:41), emphasizing his commitment to representing the people. The video visually showcases Tynkkynen's dedication through scenes of him working. The video then moves to Tynkkynen in court facing charges (Figure 2) and speaking in parliament, leaning on the podium with fumbling hands. He says: ‘I took on fines of over ten thousand euros for bringing the distress of citizens to light and spoke against the EU recovery package until my body gave in’ (0:42–0:52), highlighting sacrifices (van Knippenberg & van Knippenberg, 2005). Calm yet suspenseful, steadily intensifying instrumental music enhances a serious atmosphere (Machin, 2010). The video concludes with the text ‘SEBASTIAN TYNNKKYNEN. Your voice after elections’ as he states, ‘If you want a voice that will be heard even after the polling booths, advertisements, and politicians are gone, I'm at your service’ (0:52–1:13), highlighting his determination to continue advocating for the people (Mudde & Kaltwasser, 2017). These congruent modalities – visual (facing charges, speaking in parliament), spoken (‘I decided to use the voice’, ‘I took fines’, ‘my body gave in’, ‘I'm at your service’), music (calm), text (‘your voice’) – *co‐contextualize* each other (O'Halloran, 2008; Royce, 2007) and construct Tynkkynen as a true representative and self‐sacrificing saviour of the people (Pettersson, 2019).

## MOBILIZING A POPULIST “US”

The third identified *function* is ‘mobilizing a populist “us”’ (103), defining what ‘us’ and our values mean, and embedding shared in‐group identity by turning it into reality (Haslam et al., 2020) – in this case, within a populist framework. This function is categorized into two multimodal and discursive *strategies*: (1) celebrating ‘us’ and (2) shared victimhood.

### Celebrating ‘us’

While much populist research focuses on negative emotions (Hochschild, 2018; Wodak, 2020), less attention has been paid to how positive ones construct shared identity. As populist ideology stresses ‘the people,’ it is crucial to portray them as morally good (Ntontis et al., 2024; Sakki & Martikainen, 2021) and foster collective hope (Curato, 2016; Reicher & Haslam, 2017; Wirz, 2018).

Tynkkynen celebrates ‘us’ through positive interactive content (90). He provides information outlining the group's values and future, responds to followers' questions (56; Haslam et al., 2020), mobilizes to act or join meetups (22), and praises them (5), suggesting enticing engaged followership (Haslam et al., 2022). Most videos contain text highlighting key points (81), while in a few, it serves as the sole narrative, praising the group (4). Visually, connection with followers is constructed by speaking closely to the camera (75) and gesturing persuasively (11). Additionally, visual cues like smiling (9) and an intense colour palette create a positive atmosphere (Kress & van Leeuwen, 2021). Music also emerges as an important semiotic resource enhancing the positive mood using energetic tracks (26), including familiar melodies and pop songs. The lyrics communicate in‐group success (Abidin, 2021), and digital additions, such as emojis (10; e.g., heart), convey a sense of group excellence and group identity. To further foster a sense of belonging, Tynkkynen shares videos and images sent by followers (7). In these videos, semantically congruent modalities are set in *co‐contextualizing* relations, enhancing their communicative power (O'Halloran, 2008; Royce, 2007), emphasizing mutuality and positive in‐group energy and celebrating the in‐group, thus reinforcing the sense of ‘us’ (Haslam et al., 2020). The following example shows how in‐group celebration is constructed multimodally (Table 4).

**TABLE 4:** Multimodal transcription of Video 98.

Spoken	Visual	Music	Text
*(0–0:11)* The crowd: ‘Vote in the local elections!’	Tynkkynen stands in front of the camera wearing a checkered jacket, filming himself with a dark crowd in the background. He moves the camera to capture the entire group of followers	Joyful electronic music	‘Greetings from the Rovaniemi [city in Finland] meetup’

*Source*: https://www.tiktok.com/@sebastiantynkkynen/video/7054578712842456326 (18 January 2022, 0:11 min).

**FIGURE 3:**
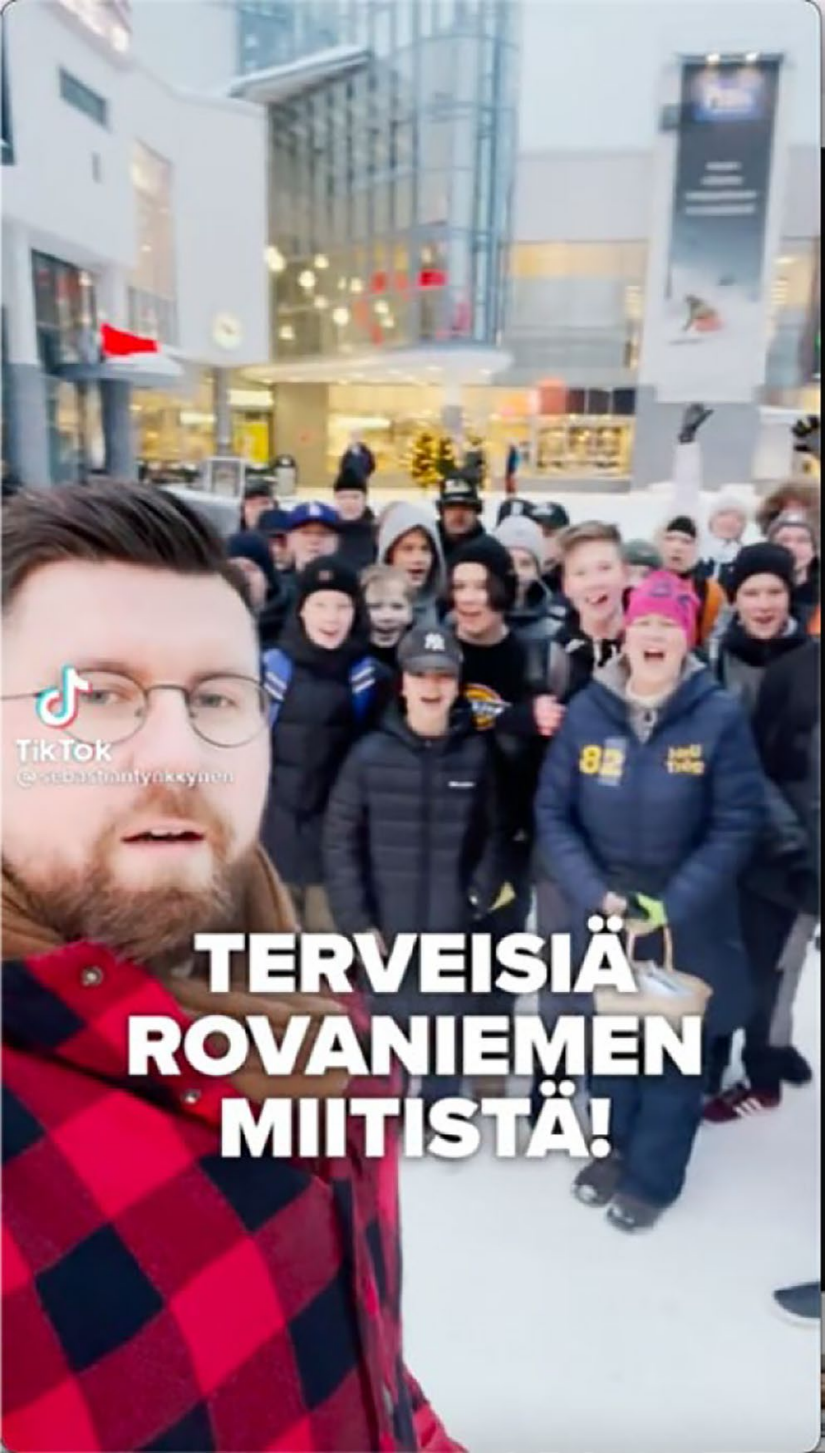
Screenshot of Tynkkynen's TikTok Video 98.

In this example, Tynkkynen stands in the foreground with a crowd behind him, highlighting his popularity (Abidin, 2021; Figure 3). The smiling crowd gives thumbs up to the camera, generating a positive atmosphere, further enhanced by joyful electronic music (Machin, 2010). The text ‘Greetings from the Rovaniemi meetup’ conveys a sense of community (Jurstakova et al., 2022). The crowd chants in unison, ‘Vote for the local elections’, not only mobilizing voting but representing a form of identity enactment (Uysal et al., 2022). Semantically congruent visual, spoken, music and text elements *co‐contextualize* each other (O'Halloran, 2008; Royce, 2007) and reinforce a sense of community and positive in‐group energy, thus fostering a shared sense of ‘us’.

### Shared victimhood

Besides celebrating the in‐group, populist leaders cultivate shared grievances and a narrative of victimhood, portraying the in‐group as collectively wronged (Mols & Jetten, 2014; Reicher & Uluşahin, 2020). This narrative strengthens group cohesion by fostering a shared purpose (Mols & Jetten, 2014) and depicting members as virtuous (Ntontis et al., 2024).

Tynkkynen portrays himself and his supporters as victims through dramatized videos (13) with congruent modalities *co‐contextualize* each other, intensifying meanings (O'Halloran, 2008; Royce, 2007), collectively communicating a victimized position and emphasizing unjust feeling, thereby rooting the narrative in shared identity (Haslam et al., 2020). He describes mistreatment directed at him or his supporters (11) as ‘political violence’ (Reicher & Uluşahin, 2020). Almost all videos include summarizing text (12) or images (e.g., news headlines) as an added digital element (4) to emphasize the narrative of mistreatment. He visually proves inappropriate behaviour encountered by recorded situations (1), adopts a serious demeanour with furrowed brows (10) and uses a dark colour palette reflecting negative emotions (Kress & van Leeuwen, 2021). A dramatic tone is also created by the lack of music.

## OTHERING THE ELITE AS ‘THEM’

The fourth identified *function* is ‘othering the elite as “them”’ (51), where the in‐group identity is formed by constructing an out‐group (Uysal et al., 2022) that does not belong and holds values different from ‘us’ (Haslam et al., 2020), reflecting the populist division between “us” and “them”. These videos are divided into two multimodal and discursive *strategies* involving (1) blaming and (2) ridiculing.

### Blaming

In populist discourse, constructing ‘them’ as morally different often occurs through blaming (Hameleers et al., 2017; Maskor et al., 2021). Research shows that provocation and hostile language towards out‐groups has been normalized in PRRP discourse (Krzyżanowski, 2020), leading to a ‘shameless normalization’ where impolite behaviour is accepted (Wodak, 2020; Wodak et al., 2021).

Tynkkynen blames out‐groups using moralized labelling (19; Ntontis et al., 2024) against Prime Minister Sanna Marin (6); political opponents (9); and Extinction Rebellion, a climate movement in Finland (4). Semantically congruent modalities that are set into *co‐contextualizing* relations (O'Halloran, 2008; Royce, 2007) reinforce each other's meanings, together communicating seriousness and that a morally bad out‐group is to blame. Speech plays a central role in these nearly 10‐min‐long videos, which all contain text underlining mocking messages. Visually, he speaks to the camera in a serious demeanour (15), emphasizing the emotional weight of the message (Hameleers et al., 2017). The absence of music further reinforces the serious tone. Also, digitally added emojis (3; e.g., angry) and red and black borders (4) serve as alerting elements conveying seriousness (Kress & van Leeuwen, 2021). To support his narratives, Tynkkynen digitally testifies to political opponents' morally bad behaviour (Mols & Jetten, 2014) using pictures and videos of them (10).

### Ridiculing

In populist discourse, insults are often veiled through visuals or irony (Sakki & Martikainen, 2021; Vuorelma, 2024). This ‘calculated ambivalence’ frames controversial messages as deliberately ambiguous and as jokes intended to be understood by the in‐group (Billig, 2001; Wodak, 2020), ridiculing and delegitimizing opponents and reinforcing in‐group superiority (Koivukoski et al., 2025; Nikunen, 2016).

Tynkkynen ironically ridicules ‘them’ (32; Koivukoski et al., 2025), targeting Prime Minister Marin, the red‐green‐left political spectrum, government (21), Extinction Rebellion, climate policy (8), feminists (1) and the media (2). Videos feature him joking or speaking ironically to the camera (13). Videos of his political opponents making nonsensical statements (6) contrast with Tynkkynen's or a fellow politician's reasonable comments (5) or sarcastic overlay text (6). Irony is visually conveyed when Tynkkynen performs or captures events without speech (13) and makes facial expressions demonstrating superiority while ridiculing others (6) with ironic text narrative (13). The videos are colourful, and some adopt a meme‐like approach (4). The ironic atmosphere is enhanced by amusing tracks (12; Abidin, 2021) coupled with digital elements like emojis (4; e.g., cry‐laugh) and filters (4), further enriching the pejorative message. Semantically congruent modalities *co‐contextualize* each other's meanings (O'Halloran, 2008; Royce, 2007), enhancing the sense of irony and ridiculing the out‐group. In contrast, divergent modalities (rational speech, facial expressions) *re‐contextualize* each other, constructing the in‐group as reasonable and establishing an ‘us’ versus ‘them’ dichotomy (Haslam et al., 2020). The following example demonstrates how ridiculing is constructed multimodally (Table 5).

**TABLE 5:** Multimodal transcription of Video 171.

Visual	Music	Text	Digital
*0:00–0:02* Tynkkynen looks at the camera with a frustrated expression, wearing a checkered shirt. He sighs and looks upwards	*X‐Files* theme	‘Greens: remove Sunday supplements’	Blue *Avatar* filter. Confused emoji
*0:03–0:09* He leans towards the camera and raises his eyebrows		‘In what reality do the Greens live?’	Frustrated emoji

*Source*: https://www.tiktok.com/@sebastiantynkkynen/video/7100581055660412165 (22 May 2022, 0:09 min).

**FIGURE 4:**
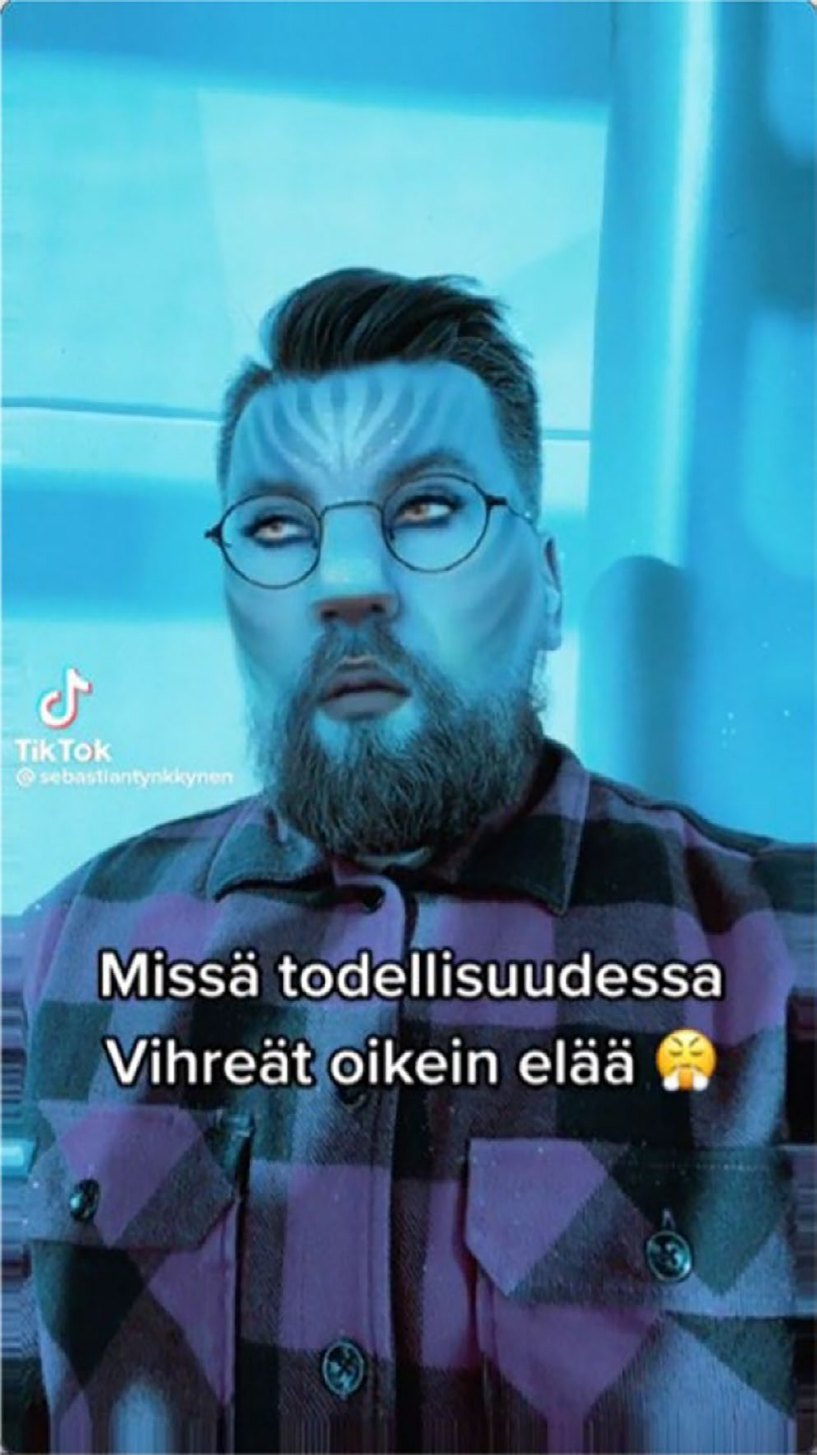
Screenshot of Tynkkynen's TikTok Video 171.

Instead of spoken narration, the video relies on other semiotic resources. Tynkkynen records himself using a filter transforming his face into a character from the fantasy movie *Avatar*, imbued with a blue hue. It operates on a memetic level, recognized as effective in political discourse (Hakoköngäs et al., 2020). The text, ‘Greens: remove Sunday supplements’, refers to the Greens Party's proposal to abolish work supplements (0:00–0:02). A confused emoji, with blushing and wide eyes, reflects Tynkkynen's emotional expression (Feng & O'Halloran, 2012). The text continues, ‘In what reality do the Greens live?’ (0:03–0:09), mockingly undermining their legitimacy (Koivukoski et al., 2025). Tynkkynen sighs and looks upward, with a frustrated emoji exhaling steam, reinforcing his irritation (Figure 4). He then leans towards the camera, taking a powerful stance (Kress & van Leeuwen, 2021). The *X‐files* theme music plays, underlying the ironic tone and suggesting conspiracies and mysteries in line with the series' theme.

Text (‘in what reality?’), music (X‐files) and digital (confused emoji, Avatar filter) elements *co‐contextualize* each other's meanings (O'Halloran, 2008; Royce, 2007), ironically highlighting the absurdity of Greens politics and their disconnect from reality, questioning whether irrational political elites can represent ‘the people’ (Pettersson et al., 2023). Meanwhile, digital (a frustrated emoji) and visual (an authoritative, irritated stance) elements are set in *re‐contextualizing* relations and position Tynkkynen as reasonable and capable of defending ‘the people’ (Mols & Jetten, 2014).

## DISCUSSION

The purpose of this study was to deepen our understanding of how populist leaders seek to construct persuasive political discourse in order to mobilize their supporters. Despite prior research, the role of social media—and the multimodal means there—has remained underexplored. The MCDP approach enabled us to uncover the diverse strategies and video‐specific affordances that populist leaders draw upon when seeking to persuade potential voters. This article explored populist persuasion through the construction of a shared social identity in social media. While there is a wide body of previous research about populism from the perspective of social identity (e.g., Bos et al., 2020; Hameleers et al., 2019; Mols & Jetten, 2014, 2016; Ntontis et al., 2024; Obradović et al., 2020), there is limited research on how populist politicians perform identity leadership and construct shared identities on social media utilizing discursive and multimodal strategies. Focusing on the most followed Finnish right‐wing populist politician on TikTok, Sebastian Tynkkynen, this article demonstrates how imaginative multimodal and discursive strategies are used to perform leadership and construct a shared identity online. Our analysis revealed eight multimodal discursive strategies used to perform leadership and construct shared identity: performing as an authentic and ordinary ‘one of us’, performing as a heroic and self‐sacrificing ‘voice of the people’, mobilizing a populist ‘us’ through in‐group celebration and shared victimhood, and othering the elite as ‘them’ through blame and ridicule.

Methodologically, the combination of rhetorical and discursive qualitative methods with multimodal approaches utilizing MCDP (Pettersson & Martikainen, 2025) allowed us to examine not only the content of the videos but also their form, and functions in detail via the language, and other semiotic elements. This three‐step analysis enabled us to identify multiple multimodal resources (Kilby & Lennon, 2021) that might not have been recognized otherwise, including discussing politics, in‐group, or personal life topics; closeups of expressions/gestures; filming events or opponents; colours; music; emojis; and added images and videos, which, down to the finest details, proved to be significant for the findings. In most strategies, multimodal resources with congruent meanings are brought into a *co‐contextualizing* relationship to create overall meanings (O'Halloran, 2008; Royce, 2007) that are exaggeratedly amplified, which makes it important to examine the modalities together. This amplification of meanings plays an important role in helping Tynkkynen to construct an image of ‘one of us’ and the ‘voice of the people’ and a shared identity, as contradictory meanings are excluded. Instead, contradictory meanings that *re‐contextualize* each other were used to create contrasts between ‘us’ (heroic and rational Tynkkynen) and ‘them’ (selfish and irrational opponents). This aligns with Pettersson et al.'s (2023) study, where different resources created an antagonistic us vs. them image of rational citizens and stupid politicians.

Theoretically, this study applies social psychological literature on populist identity leadership within the context of multimodal social media. It contributes to the application of identity leadership theory by demonstrating how the performance of leadership and the construction of social identity are digitalized and transformed within multimodal environments. Although the identity leadership model is grounded in the social identity approach (Haslam et al., 2020; Tajfel & Turner, 1979; Turner et al., 1987), the primary analytical framework of this study was MCDP (Pettersson & Martikainen, 2025), which is rooted in social constructionism. SIA perspective leans on cognitive processes underpinning political discourse to study how the leaders mobilize an essentialized social identity among the population. In contrast, CDP emphasizes the context‐dependent and flexible ways in which (social) identities are (re)constructed in talk for particular ideological and rhetorical purposes. In line with previous scholars (Burns & Stevenson, 2013; Pettersson, 2019), we argue that these perspectives are complementary, as they both focus on the construction of social identities in political discourse and persuasion and take context seriously. Multimodality/MCDP extended the CDP focus on flexible identities and their ideological functions by examining how identity leadership can be constructed and mobilized across multiple modes in a social media context.

The multimodal affordances of social media—and their interplay—enabled forms of identity leadership and social identity construction that cannot be fully captured through analyses focusing solely on verbal communication. Social media emerged in our study not merely as a communication channel, but as a *performative space of identity leadership* that offers populist leaders diverse opportunities to shape carefully curated images of themselves and to construct a “shared us” through innovative means and interaction with their followers. This, in turn, allows them to mobilize their audiences more effectively. Next, we describe how the strategies used to perform identity leadership and construct a social identity are achieved multimodally and discursively.

According to ILM, a leader's influence depends on performing as a prototypical ingroup member (Haslam et al., 2020; Rooyackers & Verkuyten, 2012). Populist leaders often present themselves as ordinary (Rapley, 1998) and authentic (Enli, 2025) to connect with ‘the people’, and this study shows how such qualities are constructed multimodally. In our analysis, ordinariness was constructed through jokes, everyday life discussions, casual clothing, smiling, cheerful colours, music, emojis and filming amusing moments – practices that can be interpreted as humanizing political figures and narrowing the distance between politicians and their followers (Beck, 2024; Wood et al., 2016). Emotions have also been identified as a central rhetorical resource in populist discourse (Hameleers et al., 2017; Wirz, 2018). In this study, emotional content – such as personal disclosures, childhood photos, closeups, wistful facial expressions, tears and emotional music – can be read as an intimate political style that signals authenticity (Bracciale & Martella, 2017; Enli, 2025).

According to ILM, populist leaders must also act ‘for us’ (Haslam et al., 2020), asserting that they are true representatives of the people (Mols & Jetten, 2014; Pettersson, 2019). Our analysis shows that populist leaders perform heroism and self‐sacrifice multimodally (Moffitt, 2016; van Knippenberg & van Knippenberg, 2005). Heroism appears in videos through speeches on political issues, straight posture, a parliament setting, chivalric music and symbolic colours. This professional image is often contrasted with clips of other MPs behaving selfishly. This narrative aligns with the populist trope of messianic leaders embodying the people's will and exposing elite hypocrisy (Moffitt, 2016). In addition, self‐sacrifice is constructed through verbal claims, direct eye contact, serious facial expressions and visual evidence of mistreatment – constructing a willingness to suffer for the group, a common populist motif (Mudde & Kaltwasser, 2017; Rooyackers & Verkuyten, 2012).

ILM frames identity leadership as a leader's ability to craft a sense of ‘us’ (Haslam et al., 2020; Reicher & Hopkins, 2001). Populist ideology relies on opposing ‘the good people’ against a corrupt elite, uniting the in‐group while fuelling contempt for the out‐group (Mudde, 2004; Mudde & Kaltwasser, 2017). This division and enacting identity is constructed through positive portrayals of ‘us’ using group values, praising, mobilizing calls, smiles, persuasive gestures, bright colours, energetic music, lyrics, heart emojis and follower‐generated content – through the increasingly common positive content on TikTok (Albertazzi & Bonansinga, 2023) as well as affective engagement and hope (Curato, 2016; Reicher & Haslam, 2017). Tynkkynen engages followers in a collective political project, similar to Trump's choreographed identity festivals, aiming to foster belonging and empowerment (Reicher & Haslam, 2017). Alongside positivity, serious videos construct a shared victimhood through narratives of mistreatment, direct camera addresses, sombre expressions, dark colours, a bowed head and filming situations of mistreatment – emphasizing shared grievances – core populist narratives (Hochschild, 2018; Mols & Jetten, 2014; Reicher & Uluşahin, 2020).

Instead, Tynkkynen constructs blame by morally labelling the culprit, emphasized multimodally through serious direct address, absent music, angry emojis and photos or videos of opponents' actions – aligning with studies highlighting accusatory rhetoric's role in emphasizing group boundaries and threats (Maskor et al., 2021). Populism thrives on fear (Mols & Jetten, 2014; Wodak, 2020), anger (Hochschild, 2018) and polarization (Mudde & Kaltwasser, 2017), and research shows that populist messages are impactful when using an identity frame portraying ordinary people as threatened by the out‐group and using emotional style while blaming (Bos et al., 2020; Hameleers et al., 2017). Tynkkynen also ridicules out‐groups ironically by joking, performing and making expressions for the camera; using colourful meme‐like clips with cheerful music and filters; and portraying opponents as irrational and himself as rational. Humour and irony have been recognized as a means of mainstreaming populist ideas by criticizing others as immoral or irrational in a seemingly ‘innocent’ manner through complex and playful messages (Pettersson et al., 2023; Wodak, 2020).

In conclusion, this article examined how Finnish right‐wing populist politician Sebastian Tynkkynen performs leadership and constructs social identity in the online spere. While this study focuses on a single Finnish politician, its findings resonate with broader international scholarship (e.g., Albertazzi & Bonansinga, 2023; Beck, 2024; Enli, 2025; Moffitt, 2016; Mols & Jetten, 2014; Mudde & Kaltwasser, 2017). However, we refrain from making broad generalizations about all politicians and all online platforms. Moreover, we do not claim that the use of the identified strategies was fully intentional; rather, we have shown that these strategies meaningfully contribute to the shaping of political discourse in contemporary digital environments. We recognize TikTok itself as a highly multimodal platform where identity performance unfolds through brief, fast‐paced videos rich in visual and auditory cues, often aligned with current trends and designed for wide circulation via algorithmic amplification, thereby facilitating continuous identity re‐performance and communal reinforcement. Future research could explore how these identified leadership strategies operate across diverse political contexts and digital platforms. We also cannot, based on this study, determine how Tynkkynen's identity leadership was received or how the analysed videos may have mobilized viewers. Future research could examine which elements of such videos have the strongest impact on audiences: whether it is the multimodality – the interplay of various modalities – that enhances engagement, or whether verbal content alone, or a particular combination of certain other modalities, is sufficient to generate persuasive effects. Such investigations would provide more detailed insight into which forms of communication on social media are most effective in mobilizing audiences.

The goal of this study was to unpack the discursive and multimodal construction of populist social identity leadership, offering new insights into how such leadership is performed and communicated online. It contributes to the social psychology of populist identity leadership on multimodal social media.

## AUTHOR CONTRIBUTIONS

**Jenni Jaakkola:** Writing – review and editing; writing – original draft; conceptualization; methodology; data curation; formal analysis. **Inari Sakki:** Conceptualization; methodology; writing – original draft; writing – review and editing; supervision; formal analysis. **Eemeli Hakoköngäs:** Writing – original draft; writing – review and editing; supervision; conceptualization; methodology. **Jari Martikainen:** Conceptualization; methodology; supervision; writing – original draft; writing – review and editing.

## FUNDING INFORMATION

This research was funded by the University of Helsinki, The Academy of Finland (Grant number 332192) and Kone Foundation (Grant number 201906612).

## CONFLICT OF INTEREST STATEMENT

The authors declare that they have no conflict of interest.

## ETHICS STATEMENT

TikTok has granted permission for the research through the TikTok Research API. The study was conducted in accordance with TikTok's ethical principles. In this study, which involves internet‐mediated research, the APA Code of Ethics and the British Psychological Society's (2017) guidelines were also followed to maximize benefits and minimize harm. Videos publicly available on TikTok were selected for analysis because they were produced by a politician, who can reasonably assume being observed due to his position of power and public role, and because the material is publicly available without the need for registration. Therefore, the potential disruption or harm was considered minimal.

## INFORMED CONSENT STATEMENT

No participants were recruited for this study that used publicly available material; hence no informed consent was sought.

## Data Availability

The video material used for this study is publicly available at TikTok: https://www.tiktok.com/@sebastiantynkkynen/video/7215996858181733658, https://www.tiktok.com/@sebastiantynkkynen/video/7217195004920384795, https://www.tiktok.com/@sebastiantynkkynen/video/7054578712842456326, https://www.tiktok.com/@sebastiantynkkynen/video/7100581055660412165.
